# Effect of Fiber–Matrix Interface Friction on Compressive Strength of High-Modulus Carbon Composites

**DOI:** 10.3390/molecules28052049

**Published:** 2023-02-22

**Authors:** Sarvenaz Ghaffari, Guillaume Seon, Andrew Makeev

**Affiliations:** Department of Mechanical and Aerospace Engineering, University of Texas Arlington, Arlington, TX 76019, USA

**Keywords:** Carbon Fiber Reinforced Polymer (CFRP), high-modulus carbon fiber, Polymer Matrix Composites (PMCs), interface strength, compressive strength, fiber hybridization, Scanning Electron Microscopy (SEM), in situ nanomechanics

## Abstract

Carbon-fiber-reinforced polymers (CFRPs) enable lightweight, strong, and durable structures for many engineering applications including aerospace, automotive, biomedical, and others. High-modulus (HM) CFRPs enable the most significant improvement in mechanical stiffness at a lower weight, allowing for extremely lightweight aircraft structures. However, low fiber-direction compressive strength has been a major weakness of HM CFRPs, prohibiting their implementation in the primary structures. Microstructural tailoring may provide an innovative means for breaking through the fiber-direction compressive strength barrier. This has been implemented by hybridizing intermediate-modulus (IM) and HM carbon fibers in HM CFRP toughened with nanosilica particles. The new material solution almost doubles the compressive strength of the HM CFRPs, achieving that of the advanced IM CFRPs currently used in airframes and rotor components, but with a much higher axial modulus. The major focus of this work has been understanding the fiber–matrix interface properties governing the fiber-direction compressive strength improvement of the hybrid HM CFRPs. In particular, differences in the surface topology may cause much higher interface friction for IM carbon fibers compared to the HM fibers, which is responsible for the interface strength improvement. In situ Scanning Electron Microscopy (SEM)-based experiments were developed to measure interface friction. Such experiments reveal an approximately 48% higher maximum shear traction due to interface friction for IM carbon fibers compared to the HM fibers.

## 1. Introduction

Characterization of carbon fibers and CFRPs, coupling knowledge in physics, engineering, and material science, is paramount for advancing CFRPs to improve structural performance characteristics for many engineering applications. CFRPs are well known for enabling lightweight, strong, and durable structures for aerospace, automotive, biomedical, sport, and other platforms. Recently, high-modulus (HM) CFRPs have attracted strong demand for building extremely lightweight aircraft structures with significant weight savings [1]. However, low fiber-direction compressive strength has been a well-recognized weakness of HM CFRPs, prohibiting their implementation in the primary structures. For example, Hexcel HM63/913 advanced continuous unidirectional HM carbon/epoxy tape with a 222 GPa (32.2 Msi) average $\frac{E_{\mathrm{Tensile}}+E_{\mathrm{Compressive}}}{2}$ Young’s modulus of the composite system exhibits a 1.01 GPa (146 ksi) fiber-direction compressive strength, which is much lower compared to a 1.54 GPa (224 ksi) fiber-direction compressive strength of Hexcel IM7/913 legacy IM carbon/epoxy tape with a 161 GPa (23.4 Msi) average Young’s modulus [1]. Moreover, HM carbon fibers with the highest fiber-direction 

Young’s modulus produce composites with the lowest compressive strength, e.g., Toray M55J/2500 unidirectional HM carbon/epoxy tape composite system with a 276 GPa (40 Msi) average Young’s modulus has only a 0.889 GPa (129 ksi) fiber-direction compressive strength [1]. The HM carbon fibers referenced in this work have 441–538 GPa (64–78 Msi) Young’s modulus while IM carbon fibers have 276–324 GPa (40–47 Msi) Young’s modulus [1].

It is worth noting that all IM and HM carbon fibers considered in this work are produced from polyacrylonitrile (PAN) precursor material. Accordingly, the fiber-direction compressive strength of the PAN-based continuous carbon fiber-reinforced polymeric tape composites is governed by microstructural stability, unlike that of pitch-based HM carbon fibers exhibiting a longitudinal-splitting failure mode. Experiments developed in Ref. [2] demonstrated kink-band formation evidencing the shear microbuckling phenomenon causing fiber-direction compressive failure in the PAN-based HM CFRPs, similar to the IM CFRPs.

**Figure 1:**
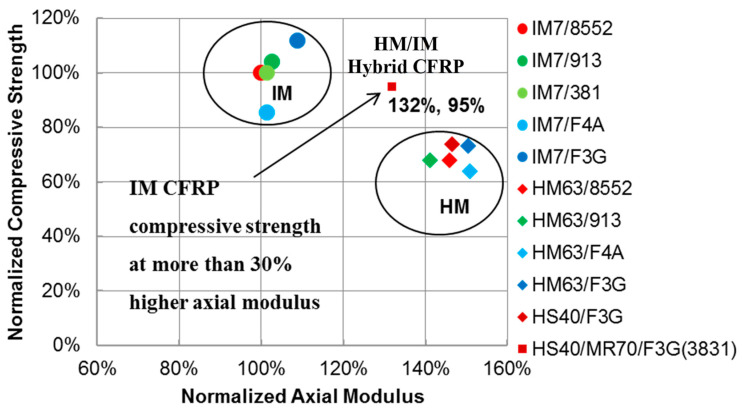
Axial modulus and fiber-direction compressive strength of HM and IM carbon-fiber-reinforced epoxy-matrix tape composites [1,2].

Microstructural tailoring may provide an innovative means for breaking through the fiber-direction compressive strength barrier of HM CFRPs. Hybridizing various fiber types has been used in the past, mostly to enhance tensile modulus and strength, and improve durability and damage tolerance [3,4,5,6]. Reference [1] demonstrated that significant improvement in the fiber-direction compressive strength of HM carbon-fiber composites may be achieved by hybridizing IM and HM carbon fibers in HM CFRP toughened with nanosilica. In particular, a new HM material solution achieving fiber-direction compressive strength of IM composites but with a more than 30% higher axial modulus has been developed [1]. Figure 1 compares the axial modulus and fiber-direction compressive strength of the new hybrid HM CFRP with various HM and IM carbon-fiber-reinforced epoxy-matrix tape composites.

The new hybrid material system is denoted as HS40/MR70/F3G(3831) in Figure 1. This prepreg composite had Mitsubishi HS40 and MR70 fibers hybridized at a 50/50 ratio and Patz Materials and Technologies (PMT) F3G 250 °F curing epoxy resin toughened with nanosilica (40% nanosilica by resin weight). F3G is equivalent to 3M 3831 nanosilica-toughened epoxy. The resin content was approximately 35% by weight (60% fiber volume fraction) [1]. HS40 is a 12K-filament-count-tow 455 GPa (66 Msi) modulus HM carbon fiber and MR70 is a 12K-filament-count-tow 324 GPa (47 Msi) modulus IM carbon fiber [1]. In addition, Figure 1 lists tape composites with Hexcel IM7 12K-filament-count-tow IM carbon fibers and HM63 12K-filament-count-tow HM carbon fibers. The resin systems include Hexcel 8552–350 °F curing toughened epoxy; Hexcel 913, Solvay 381 250 °F curing toughened epoxies currently used in aircraft structures; and PMT F4A 250 °F curing untoughened low viscosity epoxy that forms F3G after nanosilica dispersion. All CFRPs listed in Figure 1 had a similar fiber volume fraction. The material performance in Figure 1 has been normalized to Hexcel IM7/8552—a benchmark 350 °F curing prepreg tape composite material system for aircraft structures.

Hybridizing IM and HM fibers at the filament level, in addition to the matrix nano-sized structural reinforcement throughout the composite, has improved microstructural stability governing the fiber-direction compressive strength behavior. The new material solution almost doubles the compressive strength of the HM CFRPs, achieving that of the advanced IM CFRPs currently used in airframes and rotor components, but with a much higher axial modulus. The fiber-dominated axial modulus of the new hybrid composite system is exceeding the modulus of the benchmark IM CFRP by 32% due to the presence of HM fibers with significantly higher modulus compared to IM fibers as listed in the previous paragraph and in Figure 1 [1,2]. It is worth noting that a filament-level fiber hybridization has been essential as the conventional ply-level hybridization does not improve the fiber-direction compressive strength performance. To appreciate the hybridization scales, a HM and IM carbon fiber diameter is approximately 5 μm while a typical CFRP-cured ply thickness is 125–180 μm [1].

Reference [7] recognized the fact that IM carbon fibers have a significantly stronger fiber–matrix interface compared to the HM carbon fibers, governing the compression strength improvement of the new material system. The results supported the idea of integrating off-the-shelf IM carbon fibers with higher fiber–matrix interface shear strength into HM CFRPs to improve their compressive strength. This work attempts to answer the next logical question—why do IM carbon fibers have a stronger interface compared to HM carbon fibers? Fiber surfaces will be examined and compared using scanning electron microscopy (SEM) in the following section. Subsequently, in situ SEM-based experiments will shed light on the effects of the fiber surface topology differences.

## 2. IM and HM Carbon Fibers

Carbon fibers can be produced from different precursor materials, e.g., PAN, pitch, and rayon. The final properties of the fibers depend mainly on the precursor material and their structure can be controlled by changing the chemistry of the precursor or modifying the process or the conditions used to form the precursor [8,9,10,11,12]. The Young’s modulus of carbon fibers is mainly determined by the orientation and dimension of crystallite [13,14]. Increasing the precursor molecular orientation, applying tension during processing, and, most importantly, high-temperature treatments can result in the carbon fiber modulus increase.

As noted in the previous section, all IM and HM carbon fibers considered in this work are PAN-based. Manufacturing of such fibers consists of polymerization, stabilization, carbonization, and graphitization. Graphitization is the heat-treatment process to modify the properties (physical and chemical) of carbon materials using ultra-high-temperature heating (above 2000 °C) or hot stretching [15,16,17]. Higher-temperature graphitization used to produce HM carbon fibers makes the crystal structure of carbon fibers much more ordered. It helps forming a regular three-dimensional graphite crystal structure within the fiber from a random graphite sheet structure, leading to a significant increase in the tensile modulus [14,16,18]. Reference [10] conducted a series of high-temperature stretching experiments on PAN-based carbon fibers and the results showed a substantial increase in the fiber modulus with high-temperature treatments. It has been reported that increased modulus is the result of improved texturing which occurs during the heat treatment, even if the fibers are not stretched. Moreover, similar results have been observed in Refs. [17,19], experimentally confirming that increasing the heat-treatment temperature improves the fiber tensile modulus.

**Figure 2:**
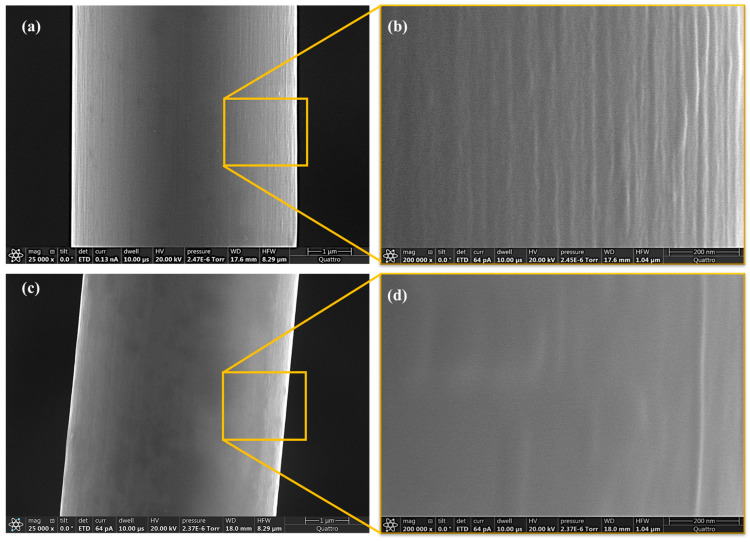
SEM images of the surface of the fiber (**a**,**b**) IM carbon fiber MR70, (**c**,**d**) HM carbon fiber HS40.

To investigate the microstructure of IM and HM fibers as a potential factor affecting the interface shear-strength measurements, the fiber surface was characterized using SEM. The results reveal a profound difference in the surface topology of IM and HM fibers. Figure 2a,b shows well-defined longitudinal grooves on the surfaces of the IM fiber. Unlike the ridged surface of the IM fibers, reflecting the structure of the polymeric precursor caused by the wet spinning process during carbon fiber manufacturing, HM fibers have a relatively smooth surface and longitudinal grooves are less noticeable, as shown in Figure 2c,d. A possible reason for the surface difference between the fibers is the higher-temperature graphitization used in the manufacturing process of HM fibers. High-temperature heat treatment can affect the morphology of fibers by increasing the preferred orientation and dimension of crystallite. Graphitic crystallites, as a main constituent of carbon fibers, are composed of layers of graphite basal planes positioned in a layered structure. Crystallites themselves are arranged in a fibrillar structure approximately parallel to the fiber axis. Increasing the heat-treatment temperature improves the size of the crystallites, modifies the distribution morphology of crystallites, and makes the axial alignment of graphitic basal planes more perfect and parallel to the fiber axis [15,19]. Accordingly, the grooves on the surface of the fiber begin to spread out, resulting in a smoother surface finish [17,20]. This observation is consistent with the results of X-ray diffraction (XRD) studies performed in Refs. [15,19,21,22,23,24] for determining the crystallite characteristics of carbon specimens. The XRD results showed that increasing the heat-treatment temperature enhanced the axial preferred orientation of the graphitic basal planes and crystallite size, indicating an improvement in the fiber graphitization degree. This information is important when relating the crystallite characteristics to the mechanical properties of the material. It is worth noting that Young’s modulus, as an inherent property of carbon fibers, can be directly affected by changes to the microcrystalline structure of the fiber. By increasing the crystallite size, degree of the preferred orientation, and the surface graphitization degree, Young’s modulus of the fiber can be substantially increased [19,20,21,25].

Surface roughness can significantly affect fiber–matrix interface behavior. Schadler et al. [26] recognized roughness as the most important factor for improving the interfacial shear strength of carbon-fiber–thermoplastic composites. Surface roughness of the fiber can increase interfacial friction by improving micromechanical bonding (interlocking) of the fiber and matrix surfaces that are in contact. In addition, it allows the matrix material to better penetrate available crevices in the fiber surface and increase the interpenetration of both materials [27,28,29]. Furthermore, a larger surface area of the fiber can increase the potential for chemical bonding [27].

## 3. In Situ SEM-Based Experiments

**Figure 3:**
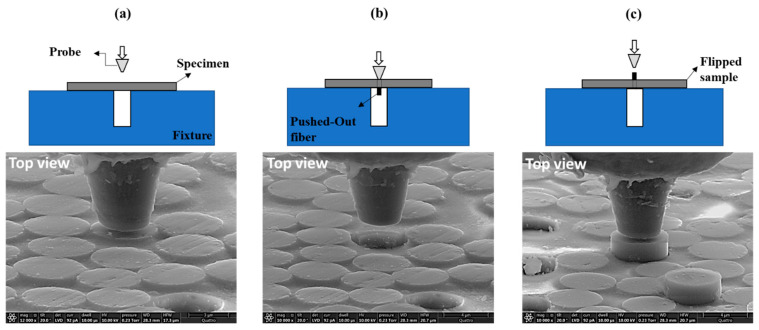
Schematic and SEM images of the test set-up: (**a**) Before push-out; (**b**) After push-out; (**c**) Before push-back.

The major focus of this work has been understanding the fiber–matrix interface properties governing the fiber-direction compressive strength improvement of the hybrid HM CFRPs. In particular, differences in the surface topology may cause much higher interface friction for IM carbon fibers compared to the HM fibers, which is responsible for the interface strength improvement. For reference, Jero [30,31], Parthasarathy [32] and Carter [33] showed the importance of surface roughness on the interface frictional behavior of ceramic composites. They conducted fiber push-back tests to move the previously pushed-out fibers back through the material and addressed the contribution of interface topography on the sliding friction. It is worth noting that in situ SEM tools to image the deformation and failure process during the experiments were not available at that time. In this work, in situ SEM-based fiber push-out and push-back tests are utilized for a hybrid CFRP to measure HM and IM carbon-fiber–polymer-matrix interface shear strength and interface friction strength, respectively. A micromechanical load frame integrated with SEM is used to debond the fiber–matrix interface and push individual fibers out of a thin membrane. After the push-out, protruding the end of the fiber from the sample, the sample is turned over and pushed-out fibers are reloaded in the reverse direction and moved back into the matrix. Figure 3 shows schematic and the SEM images of the fiber push-out and push-back experiments.

## 4. Sample Preparation

In preparing the specimens for a push-out test, a thin slice of approximately 5 mm × 5 mm × 0.5 mm was cut from a unidirectional composite panel with a precision high-speed diamond saw. A sample surface perpendicular to the fibers was polished with silicon carbide abrasive papers of 320, 600, 800, and 1200 grit. Then, the polished surface was placed on a disk grinder using a Crystal Bond adhesive. To achieve the final sample thickness of 20–30 μm and create a plane parallel sample surface perpendicular to the fiber direction, the opposite surface of the sample was thinned out using a lapping process following the same series of silicon carbide papers and finished with a colloidal silica suspension of 0.04 μm. To remove the sample from the polishing device, the disk grinder was heated up to 120–140 °C and the specimen was slid off the grinder with a razor blade. Finally, the sample surfaces were carefully cleaned with an acetone-soaked cotton swab to remove any residue of the adhesive.

**Figure 4:**
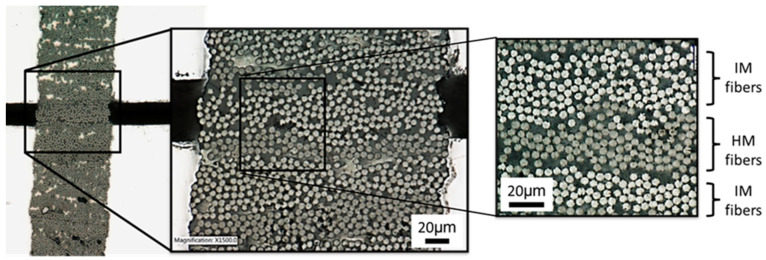
Micrographs of the sample surface generated using a white-light optical microscope at 500 and 1500× magnification.

Next, optical microscopy was used to identify and map IM and HM fibers on the polished sample surface for the in-situ SEM testing. It should be noted that HM and IM fibers are identical in diameter and surface morphology. However, their different response to light reflection/absorption results in dissimilar colors in the micrograph and enables us to distinguish the HM and IM fibers (Figure 4). The sample was placed on a steel fixture with a 50 μm wide groove engraved on its surface. The area that contained both types of fibers was positioned over the groove and the edges of the sample were taped down to the fixture using conductive tape to ensure close contact to the substrate. All the experiments discussed in this study were carried out on the same hybrid sample with a thickness of 23 μm.

## 5. Test Procedure

In this study, push-out and push-back experiments were performed in a PI-88 Nanomechanical load frame (Bruker, Eden Prairie, MN, USA) with a 4 μm diameter flat-end diamond indenter tip. A 3-plate capacitive transducer technology in the system provides high sensitivity for force measurement and a piezo-based flexure controls displacement measurement and actuation [34]. The tests were performed in displacement control to enable the measurement of load drops with a displacement rate of 30 nm/s. It is worth noting that the maximum indenter displacement without touching the matrix depends on the tip geometry and the fiber radius. With the assumption that the indenter tip is positioned centered on the fiber and the surrounding matrix does not deform, the maximum allowed indenter displacement of 3 μm was calculated for the flat-end tip being used in this study. The fixture was placed inside the SEM, and the indenter tip was brought into contact with the fiber. IM and HM fibers placed above the groove were selected strategically to be loaded. The force applied on the fiber leads to fiber–matrix interface debonding until the fiber completely loses its load-carrying capacity and protrudes from the opposite sample surface. During the test, the load and depth of indentation are continuously recorded and the interface shear strength (*IFSS*) is calculated using the maximum applied load *P_max_*, fiber length (the thickness of the specimen) *h*, and fiber radius *r*.

(1) $$IFSS=\frac{P_{max}}{2\pi rh}$$

**Figure 5:**
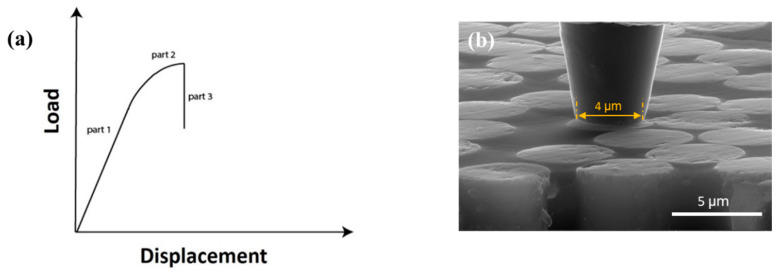
(**a**) Schematic of the load–displacement response expectation for a single fiber push-out test; (**b**) flat-end diamond tip.

A typical load–displacement response expectation for a single-fiber push-out test is shown in Figure 5a. It presents an initial linear elastic loading zone associated with the elastic deformation of the fiber and surrounding matrix when a perfectly bonded interface exists, followed by a non-linear region which represents the beginning of interface failure. As interface fracture progresses slowly with increasing load, compliance increases until the maximum load has been reached. At this stage, failure propagation along the interface becomes unstable and the fiber push-out will initiate. Full debonding of the interface can be characterized by an abrupt load drop on the load–displacement curve (part 3). It is worth noting that after the complete interface debonding, the probe is almost touching the matrix. Accordingly, after the fiber–matrix interface has been completely debonded and individual fibers are pushed out of the thin membrane, the test sample is turned over and pushed-out fibers are reloaded in the reverse direction to assess friction. By measuring the maximum applied load *P_max_* during the push-back test, the thickness of the specimen *h*, and the fiber radius *r*, the fiber–matrix interface friction strength can be calculated using Equation (1). To ensure adequate statistics, six fibers were tested for each fiber type.

## 6. Results

**Figure 6:**
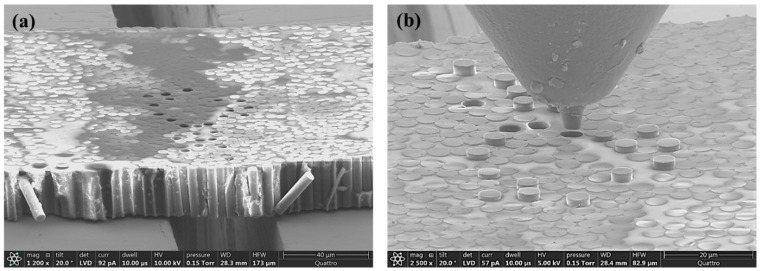
SEM observation of the pushed-out tested specimen: (**a**) top view and (**b**) back view of the pushed-out carbon fibers.

The top and back side of the tested sample were observed with SEM after push-out tests, and micrographs are shown in Figure 6. It can be seen from both the top (Figure 6a) and the back sides (Figure 6b) that the tested fibers were pushed out successfully.

**Figure 7:**
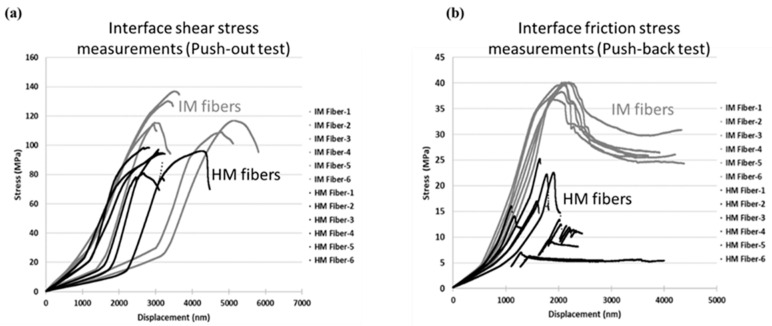
Comparison of the stress–displacement curves for IM and HM fibers; (**a**) push-out test configurations to measure interface shear stress; (**b**) push-back test configurations to measure interface friction stress.

Figure 7 summarizes the resulting stress–displacement curves for IM and HM carbon fibers in hybrid composite. The black curves represent HM fibers and gray curves determine IM fibers. When it comes to *IFSS* measurement from the fiber push-out tests, Figure 7a confirms that the average measured *IFSS* value in IM fibers (122 Mpa) is 30% higher than HM fibers (93 Mpa). It verifies that IM fibers have a stronger interface compared to HM fibers [7,35]. Compared to Figure 5a, Figure 7a reveals a large scatter (displacement shift) in the initial part of the stress–displacement response of individual fibers caused by poor contact between the sample and the test fixture, potentially due to imperfectly flat surfaces causing contact imperfections. The poor contact conditions may also result in rigid body motion, especially in the beginning of the push-out test, creating compliance artifacts [36]. To reduce artifacts in the load–displacement response, the sample-preparation process can be improved. However, due to the damaging nature of the polishing/lapping process used to create such thin samples, the artifacts cannot be completely excluded.

Furthermore, the results of the study to compare fiber–matrix interface friction stress for both IM and HM fibers are presented in Figure 7b. According to the outcome of the fiber push-back tests, the average interfacial friction strength of HM fibers was estimated as 18.9 MPa while IM fibers in the same material system have an average interfacial friction strength of 36.7 MPa. The test results show an approximately 48% decrease in the average values of the fiber-to-matrix interface friction strength for the HM carbon fibers compared to their IM counterparts. The 18 MPa difference in the interface friction between IM and HM fibers appears to have a major contribution to the 29 MPa *IFSS* difference between IM and HM fibers. In particular, a substantial difference in fiber surface topology, as illustrated in Figure 2, with IM fibers exhibiting much more surface roughness compared to HM fibers, is likely to be an underlying cause.

## 7. Concluding Remarks

This work advanced fundamental knowledge and understanding of the fiber–matrix interface properties governing the fiber-direction compressive strength improvement of hybrid HM CFRPs. Fiber–matrix interface friction was investigated as a potential mechanism driving the improvement of the interface shear strength for IM fibers compared to HM fibers. First, the microstructure of the fibers was examined using SEM. Images of the surfaces of Mitsubishi MR70 IM and HS40 HM carbon fibers reveal grooves on the IM fibers, which can affect fiber–matrix adhesion and interfacial friction performance compared to HM fibers with smooth surfaces. Next, in situ SEM-based experiments were performed to measure the interface shear strength and interface friction to shed light on the effects of the fiber surface topology differences. Such experiments reveal an approximately 48% higher maximum shear traction due to interface friction for IM carbon fibers compared to HM fibers. The difference in the interface friction—18 MPa appears to have a major contribution to the 29 MPa interface shear strength difference. This work showed that in situ nanomechanics-based (SEM data-driven) experimental methods measuring the essential microstructural properties, including fiber–matrix interface shear strength, and measuring the contribution of various components such as friction are key to explaining why IM carbon fibers may have a stronger interface compared to HM carbon fibers.

## Data Availability

Not applicable.
